# Subcutaneous Ertapenem and Temocillin for Symptom Control of Pelvic Collection in the Palliative Care Setting: A Case Report

**DOI:** 10.7759/cureus.114298

**Published:** 2026-08-10

**Authors:** Oliver Maynard, Christopher Nobbs

**Affiliations:** 1 Palliative Medicine, London North West Healthcare NHS Trust, Southall, GBR

**Keywords:** ertapenem, pelvic collection, subcutaneous antibiotics, symptom control, temocillin

## Abstract

Infections can significantly impair quality of life in patients with life-limiting disease, yet antibiotics are infrequently used for symptom control in palliative care due to challenges in administration, concerns about antimicrobial resistance, and limited clinical guidance. Emerging evidence and pharmacokinetic data support the subcutaneous route as a viable option for delivering several antibiotics.

We present a female patient in her 50s with an incurable presacral collection secondary to rectosigmoid adenocarcinoma who presented with fever, pain, and rectal discharge refractory to initial management. Subcutaneous ertapenem, followed by temocillin, resulted in marked symptomatic improvement and was well tolerated with infusion rate adjustments.

This intervention led to a significant improvement in quality of life, enabling over four months at home and the achievement of important personal milestones.

This case highlights the potential role of subcutaneous antibiotics for symptom control in palliative care and underscores the need for further consensus guidance.

## Introduction

Infections are common in patients with advanced cancer and at the end of life and can cause significant symptom burden [1,2]. Despite this, there is limited evidence and no clear consensus guidance supporting antibiotic use for symptom control in palliative care [1-4]. However, emerging evidence suggests that antibiotics may improve quality of life in selected patients in the last stages of life [5-7]. 

In this population, oral administration is often not feasible and intravenous therapy can present challenges in terms of vascular access and appropriate care setting [8,9]. Subcutaneous administration provides a potential alternative route of antibiotic delivery and is supported by pharmacokinetic studies demonstrating efficacy [10,11]. The Palliative Care Formulary (PCF) provides guidance on antibiotics that may be administered subcutaneously [12]. 

Decisions regarding antibiotic use in life-limiting illness should be individualised, with the potential symptomatic benefit balanced against the risk of adverse effects and the potential to escalate antimicrobial resistance [4]. 

This report aims to describe the successful use of subcutaneous antibiotics for symptom control in a patient with an infected malignant presacral collection receiving palliative care, specifically ertapenem and temocillin which were selected for their benefit against multidrug-resistant organisms, and supportive evidence of safe administration in the PCF. This report highlights the practical considerations, tolerability, and potential quality-of-life benefits of subcutaneous antibiotic administration when oral and intravenous routes are unsuitable.

## Case presentation

A woman in her 50s was diagnosed with adenocarcinoma of the rectosigmoid junction after presenting with rectal bleeding. Initial management included a laparoscopic defunctioning loop colostomy, followed by neoadjuvant chemoradiotherapy. She subsequently underwent anterior resection with formation of a defunctioning loop ileostomy. 

**Figure 1:**
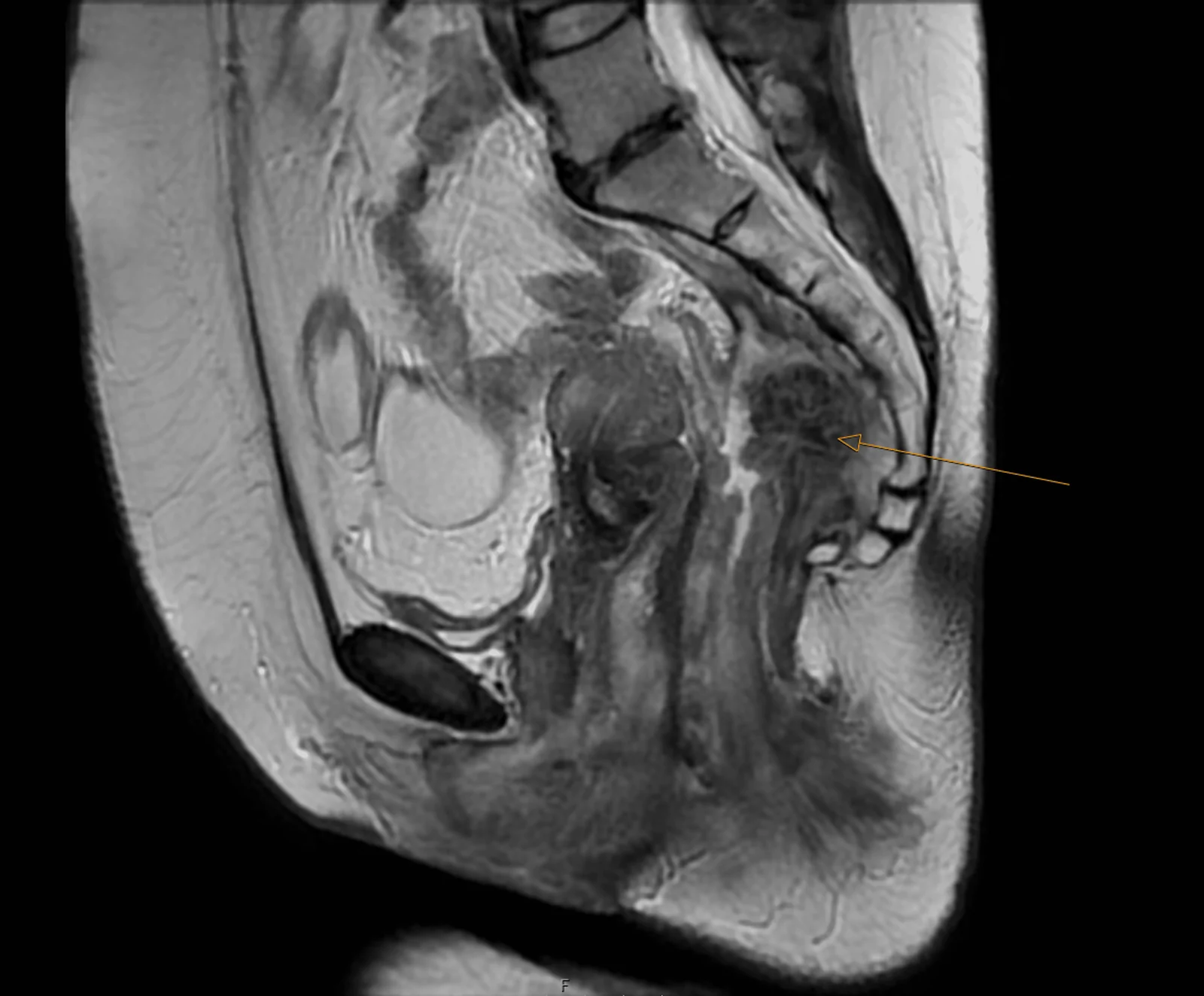
Sagittal pelvic MRI demonstrating a presacral collection at the colorectal anastomotic site (arrow)

Approximately one year later, she developed purulent rectal discharge. Endoscopic assessment confirmed disease recurrence and magnetic resonance imaging demonstrated a presacral collection at the anastomosis with marrow signal changes in the sacrum concerning for osseous involvement (Figure 1). 

She received several lines of palliative chemotherapy, which were discontinued as her disease progressed. With no further oncological or surgical treatment options available, her management transitioned to best supportive care. 

The patient experienced persistent 9-out-of-10 abdominopelvic pain and high-volume purulent rectal discharge occurring more than 10 times daily, which significantly impaired her quality of life. Surgical interventions, including endo-sponge placement and intraoperative washout with clipping, were deemed unsuitable due to friable tissues. The patient required multiple admissions to the specialist palliative inpatient unit (SPIU) for management of pain, infective symptoms, and rectal discharge. 

Differential diagnosis 

The patient’s symptoms were primarily attributed to an infected malignant presacral collection. The presence of purulent discharge, radiological findings (Figure 1), and microbiological results supported this diagnosis. 

Alternative causes of fever and systemic symptoms, including tumour-related pyrexia, were considered. However, the clinical response to antibiotic therapy and associated biochemical improvement supported infection as the predominant contributor to her symptoms. 

Timeline of admissions

Table 1 shows the events of the patient's three admissions in chronological order.

**Table 1:** Timeline of admissions WBC: white blood cells; CRP: C-reactive protein; Neut: neutrophils; SC: subcutaneous; PO: oral; IV: intravenous; ESBL: extended-spectrum beta-lactamases; SPIU: specialist palliative inpatient unit Reference range for WBC: 3 to 10x10^9/L; reference range for Neut: 2 to 7.5x10^9/L; reference range for CRP: 0 to 5mg/L

Pre-admission in community		

	Clinical Event/Intervention	Outcome
	Hyoscine patch 1mg/72hrs	No reduction in rectal discharge volume
	Glycopyrronium SC 200mcg four times daily	No reduction in rectal discharge volume
First admission to the SPIU		
Day	Clinical Event/Intervention	Outcome
0	Worsening rectal discharge and pain	Admission to the SPIU
1	Wound management systems sought	Due to the anatomical location of the collection, no suitable system could be applied
1	Supportive measures (fan and hygiene interventions)	Provided minimal relief
0-5	Octreotide SC titrated to 400mcg daily	No reduction in rectal discharge volume
0-20	Paracetamol 1g four times daily	Temperatures >37.5c persisted
13	Rectal discharge swab and culture	Heavy Growth of *Escherichia coli *
14-16	Metronidazole PO 400mg three times daily + Ciprofloxacin PO 500mg twice daily	No improvement in symptoms
16	Antibiogram from rectal discharge culture	ESBL producer – sensitive to carbapenems
16-20	Meropenem IV 1g three times daily	Fevers resolved, and rectal discharge became less frequent and viscous
20	Discharged from the SPIU	Remained at home for approximately two months with improved symptoms
Second admission to the SPIU		
Day	Clinical Event/Intervention	Outcome
0	Fevers >39 °C and viscous rectal discharge	Admission to the SPIU
1	IV cannulation failed	The patient did not want to be transferred to the hospital for definitive IV access
2	Blood cultures	No growth
3	Repeat rectal discharge swab and culture	Mixed growth of unclear significance
5-11	Ertapenem SC 1g once daily	Fevers resolved
6	Local site reaction at the SC line port. Infusion slowed from 30 min to 2 - 4 hr	Better tolerated, with resolution of site reaction
13	Discharged from the SPIU	Further two months at home, where she was able to complete important life events
Third admission to the SPIU		
Day	Clinical Event/Intervention	Outcome
0	Pain, fevers, and rectal discharge recurrence	Admission to the SPIU
0	Bloods tests	Raised inflammatory markers (WBC 15.9x10^9^/L, Neut 13.4x10^9^/L, CRP 263 mg/L)
0-4	Ertapenem SC 1g once daily	Fever and sweats persisted
4	Bloods tests	Increasing inflammatory markers (WBC 18.5x10^9^/L, Neut 15.5x10^9^/L, CRP 271 mg/L)
4	Rectal discharge swab	Growth of *Escherichia coli *sensitive to penicillin
4-12	Temocillin SC 2g twice daily	Resolution of fevers and sweats, and less frequent rectal discharge
5	Local site irritation at SC line port. Infusion slowed from 20min to 60min	Better tolerated, with resolution of local site reaction
11	Blood tests	Improvement in inflammatory markers (WBC 14.8x10^9^/L, Neut 12x10^9^/L, CRP 118 mg/L)
13	Discharged from the SPIU	Improved quality of life

Interventions 

Ertapenem was administered once daily by reconstituting 1g of powder for solution for infusion in 10ml sodium chloride 0.9% and further diluted in 50ml sodium chloride 0.9% and delivered via subcutaneous line under gravity. The infusion was initially administered over 30-60 minutes. However, the patient experienced localised pruritus at the administration site. Slowing the infusion to 2-4 hours led to complete resolution of this adverse effect. Mild localised swelling was observed at the infusion site, without erythema or pain. The administration site was changed daily, and a seven-day course was completed, which resulted in a reduction in pain scores to 4-out-of 10, reduction in the frequency of rectal discharge to less than three times daily, and no further fevers greater than 37.5 °C. 

On symptom recurrence, subcutaneous ertapenem was retrialled owing to previous evidence of benefit, but without response. Repeat pus culture demonstrated growth of *Escherichia coli* sensitive to penicillin. Following discussion with the Microbiology team, the decision was made to change to a penicillin-class antibiotic. The PCF recommends two penicillin-based agents for subcutaneous administration: piperacillin + tazobactam and temocillin. Temocillin was selected due to lesser frequency and infusion fluid volume requirements. 

Temocillin was administered twice daily by reconstituting 2g of powder for solution for injection in 2ml of 1% lidocaine and diluting to 20ml with water for injection. It was administered via abdominal subcutaneous line using a standard infusion pump over thirty minutes. The deviation from the PCF guidance, which suggests lower infusion volume, was necessary as a precision infusion pump was not available. 

Local site irritation was noted with 30-minute infusions. Increasing the infusion duration to 60 minutes improved tolerability. A 10-day course led to no further fevers greater than 37.5 °C, cessation of rigors and sweats, and reduced rectal discharge and pain scores to 3-out-of-10. There was accompanying biochemical improvement, with reductions in white cell count, neutrophils, and C-reactive protein. 

## Discussion

Pharmacokinetic data and formulary guidance support the use of the subcutaneous route for selected agents, including ertapenem and temocillin [8,10,12]. The goal of antibiotic therapy was symptom control, as definitive resolution of the infection was deemed impossible due to lack of source control. The predefined end point of the trial of subcutaneous antibiotics was improvement in symptoms of fevers, pain, or pus volume/frequency after a 5-to-10-day course. If there was no improvement, then antibiotics would be stopped. This was discussed and agreed with the patient prior to initiation of antibiotic therapy. 

This case demonstrates the value of microbiological guidance in antibiotic selection [10,12]. Meropenem, which had a good effect intravenously in her first admission, has limited evidence supporting subcutaneous administration. This was why ertapenem was chosen when intravenous access failed, as it is in the same antibiotic class, and previous microbiology showed sensitivity to carbapenems. Also, microbiological guidance and careful selection of antimicrobial agents helped mitigate the risk of antimicrobial resistance, which was a particular concern in this case as resolution of the pelvic infection was not possible, therefore creating a greater risk of drug-resistant bacteria which may have led to already refractory symptoms becoming uncontrollable. 

Although antibiotic use in the palliative setting remains debated due to concerns regarding unpredictable benefit and antimicrobial resistance, emerging data suggest that selected patients may derive meaningful symptomatic benefit [1,5-7]. In this case, treatment resulted in a reduction of fevers greater than 37.5 °C, pain scores, and frequency of rectal discharge, allowing prolonged time at home and achievement of important personal goals. This outcome aligns with recommendations that antimicrobial prescribing in palliative care should be guided by patient-centred outcomes and quality-of-life considerations [1,3,4].

## Conclusions

Subcutaneous ertapenem and temocillin provided effective symptom relief for an infected malignant presacral collection when oral and intravenous antibiotic administration were not feasible. Treatment was associated with reductions in fever, pain, and purulent discharge, improved quality of life, and prolonged time in the patient’s preferred place of care. Infusion-related adverse effects were mitigated through optimisation of administration techniques, including longer infusion durations, regular site rotation, and close monitoring.

This case highlights the potential role of carefully selected subcutaneous antibiotics as a pragmatic, patient-centred intervention in palliative care, where symptom management and minimisation of treatment burden are key priorities. Antibiotic use should remain aligned with individual goals of care, balancing symptomatic benefit against the risks of adverse effects and antimicrobial resistance. Further research and consensus guidance are needed to inform clinical practice in this area.
